# Safe and informed prescribing of psychotropic medication during the COVID-19 pandemic

**DOI:** 10.1192/bjp.2020.92

**Published:** 2020-05-04

**Authors:** Jurjen J. Luykx, Sisco M. P. van Veen, Arne Risselada, Paul Naarding, Joeri K. Tijdink, Christiaan H. Vinkers

**Affiliations:** 1 Departments of Psychiatry and Translational Medicine, UMC Brain Center, University Medical Center Utrecht, Utrecht University; and Second Opinion Out-patient Clinic, GGNet Mental Health, Warnsveld, The Netherlands; 2 Department of Psychiatry, UMC Brain Center, University Medical Center Utrecht, Utrecht University, The Netherlands; and Department of Medical Humanities, Amsterdam UMC, The Netherlands; 3 Department of Clinical Pharmacy, Wilhelmina Hospital Assen, The Netherlands; 4 Department of Old Age Psychiatry, GGNet Mental Health, Warnsveld, The Netherlands; 5 Department of Medical Humanities, Amsterdam UMC; and Department of Philosophy, VU Universiteit, Amsterdam, The Netherlands; 6 Departments of Psychiatry and Anatomy and Neurosciences, Amsterdam UMC, The Netherlands

**Keywords:** COVID-19, psychopharmacology, prescribing, psychiatry, epidemiology

## Abstract

Treatment with psychotropic medication may sometimes be jeopardised because of the COVID-19 pandemic. One underlying reason is the lack of COVID-19-specific psychopharmacology guidelines. Here, we discuss five considerations arising from our clinical experience and pharmacological background knowledge to enable safe and well-informed psychopharmacotherapy during the COVID-19 pandemic.

Many patients’ treatments with psychotropic medication may currently be jeopardised in several ways because of the COVID-19 pandemic and subsequent policies of social distancing and lockdowns. Examples from our own experience range from a long-term clozapine user who developed severe neutropenia after contracting COVID-19, patients not willing to collect their medicines from pharmacies and elderly patients discontinuing visits to laboratories for therapeutic drug monitoring (TDM). Furthermore, in the current uncertain era of the COVID-19 pandemic, psychiatrists and clinical pharmacists resort to existing, non-specific prescribing guidelines for patients with psychiatric illnesses.

Here, we reason that, given the lack of specific guidelines in this unprecedented situation on the one hand and the existence of recently emerged COVID-19 online resources (see below) on the other, several considerations may be of use to enable safe prescribing of psychotropic medication during the current pandemic. We have divided these considerations into several categories, illustrating them with real-life clinical dilemmas and describing potential solutions to optimise patient care during this outbreak. By drawing data from COVID-19-specific online resources and clinical trial data unrelated to COVID-19 we compiled a table listing preferred psychotropic medication per drug category. As the current pandemic is affecting people without a history of mental illness as well as those with severe mental illness (SMI) we target both populations. Conceivably, people with SMI are hit harder by the pandemic owing to relatively high rates of homelessness, smoking/illicit drug use and poor general health among them.

Although we focus on psychotropic medication, it is important that psychological treatments are not overlooked. They may be just as effective for a range of symptoms that people with and without a history of mental illness may currently present with. This crisis will likely have a large psychological impact on individuals both with and without a history of mental illness, resulting in anxiety, excessive worrying and insomnia in many. Such symptoms may be readily treated by psychological interventions, including (online) cognitive–behavioural therapy and other forms of psychotherapy. Thus, even though the focus of this piece is on pharmacotherapy, clinicians should be aware of the pivotal role of psychological treatments during this pandemic. Moreover, we believe that our recommendations about safe and informed prescribing of psychotropic medication can help identify clinical situations (e.g. a patient with a history of prolonged QT interval) where psychological treatments may be the only viable option.

## New-onset psychiatric problems and reduced access to usual care

First, the virus itself can bring about a range of challenges for physicians and patients as the uncertainties surrounding it endanger the safe use of psychotropic medication. This is a pressing problem as recent evidence suggests that anxiety, insomnia, use of psychotropic medication and drug misuse are rising during the current pandemic,^1,2^ resulting in an important discrepancy: safe prescribing of psychotropics has become both more challenging and more needed. People with new-onset anxiety and insomnia may benefit from a short-term prescription of a sedative or anxiolytic, but real-life out-patient consultations have become more challenging as out-patient facilities try to restrict elective care and face-to-face contacts. In addition, follow-up consultations are currently hampered for those patients who experience difficulties communicating by telephone, resulting in a lack of clarity about collecting prescriptions.

Another example of problems that have emerged is (perceived) restricted access to laboratories for elderly patients. One of our elderly patients recently decided to stop going to a laboratory for her regular TDM of lithium to reduce the likelihood of COVID-19 transmission. Importantly, as an older person she is at increased risk of suffering both the psychosocial and physical consequences of COVID-19 infection, ranging from loneliness to developing fever, dehydration and thus, potentially, lithium toxicity. As an out-patient, she is not in constant touch with her care team and, since she lacks the ability to engage in video calling, encouraging her to go to the laboratory has become more cumbersome. In these times, if TDM is absolutely necessary, we should consider the option of nurses or physicians calling at these patients’ homes to draw blood since several in-patient and out-patient psychiatry clinics are currently less busy. Clearly, during such visits we should aim to wear protective gear and keep a distance of at least 1.5 m as much as possible. For clozapine, we should consider speeding up the use of dried blood spots for TDM, as this has proven validity.^3^

## Psychotropic–COVID-19 drug interactions and COVID-19 drug side-effects

Second, interactions between psychotropic medication and COVID-19 medication (atazanavir, lopinavir/ritonavir, remdesivir, favipiravir, (hydroxy)chloroquine, interferon beta, ribavirin and tocilizumab) can be serious. The University of Liverpool provides an overview of possible interactions with COVID-19 medication, including psychotropics (www.covid19-druginteractions.org). These interactions are divided into increased and decreased exposure (measured by blood levels of medications) and QT and/or PR interval prolongation. We note that in most instances people requiring COVID-19 medication are hospital in-patients with electrocardiogram (ECG) monitoring possibilities. Furthermore, since most psychotropic drugs are partly metabolised by CYP3A4, concomitant use of atazanavir or lopinavir/ritonavir may increase their plasma levels owing to CYP3A4 inhibition. However, this will not always lead to more side-effects since some psychotropic drugs (e.g. selective serotonin reuptake inhibitors) do not carry dose–response relationships.

Besides interactions we should also be wary of COVID-19 medication with high risks of psychiatric adverse drug reactions. Mefloquine, for example, carries a relatively high likelihood of neuropsychiatric side-effects, ranging from agitation to psychosis.

Although more studies should be conducted to explore the interactions between psychotropic medication and COVID-19 medication, several observations may help clinicians choose a drug in new patients requiring psychopharmacological treatment. Importantly, as we are currently uncertain how many people will contract the virus in the near/far future and therefore are unsure who will need COVID-19 medications, such considerations may apply to all new psychiatric patients currently seen by physicians. In addition, we caution against discontinuing or deciding against efficacious psychotropic drugs such as lithium and clozapine out of fear of interactions. On the basis of the above-mentioned considerations and background knowledge of psychopharmacological profiles, in Table 1 we summarise which psychotropic drugs may be preferable during experimental COVID-19 therapies and which psychotropic medication should be prescribed with caution.

**Table 1:** Preferred psychotropic drugs during experimental COVID-19 therapies

Drug class	Caution for QT and/or PR interval prolongation^a^	Caution for drug–drug interactions^b^	Preferred drugs^c^
Antidepressants	(Es)citalopram Mirtazapine Tricyclic antidepressants^d^ Venlafaxine	St John's wort	Agomelatine Bupropion Duloxetine Fluoxetine Fluvoxamine Paroxetine Sertraline
Antipsychotic drugs	All, except for: aripiprazole, brexpiprazole, cariprazine, lurasidone	Pimozide Quetiapine	Amisulpride^e^ Aripiprazole Brexpiprazole Cariprazine Lurasidone Olanzapine^e^
Benzodiazepines	None	All (especially midazolam), except for: lorazepam, lormetazepam, oxazepam, temazepam^f^	Lorazepam^f^ Lormetazepam^f^ Oxazepam^f^ Temazepam^f^
Mood stabilisers	Lithium	Carbamazepine	Lamotrigine Lithium^e^ Valproate

a. Relevant when lopinavir/ritonavir or (hydroxyl)chloroquine are used to treat COVID-19.

b. Especially relevant when atazanavir or lopinavir/ritonavir are used to treat COVID-19, because CYP3A4 inhibition causes higher blood levels of psychotropic drugs. St John's wort and carbamazepine may reduce blood levels of several drugs used to treat COVID-19.

c. Therapeutic drug monitoring may be helpful when considering dose modifications.

d. For example amitriptyline, clomipramine, imipramine, nortriptyline.

e. Based on efficacy, with careful monitoring of electrocardiogram if applicable.^4^

f. For sedatives/anxiolytics/hypnotics, the benzodiazepines with no active metabolites that only undergo glucuronidation (lorazepam, lormetazepam, oxazepam, and temazepam) are generally preferred.

## Adjusting psychotropic prescribing to reduce clinic visits

Third, social distancing is bringing about a number of changes in the way we and our patients relate to our environments. As psychiatrists, we should be proactive and, for patients with SMI, consider switching to compounds with a longer half-life if possible. For example, a patient on paliperidone 1-monthly injections may be switched to paliperidone every 3 months if they have been stable for over 4 months, thus reducing the likelihood of contracting COVID-19 for nurses. Similarly, for new patients, longer half-life antidepressants such as fluoxetine may be preferred as discontinuations may result in less severe withdrawal symptoms and less fluctuations in TDM. Furthermore, in the event of equipoise (equal effectiveness) we may currently consider prescribing agents that do not require TDM.

## Psychotropic prescribing for COVID-19-positive patients

Fourth, we should consider that possible consequences of the disease in COVID-19-positive patients should influence how we prescribe medication. A study from China reports a 20% incidence of neutropenia in COVID-19-positive patients,^5^ although neutropenia may also be observed in those testing positive for influenza.^6^ In support of this observation, one of our patients who had been on clozapine for many years and had tested positive for COVID-19 suddenly suffered a severe drop in neutrophil count. Although currently unknown, possibly COVID-19-positive patients on clozapine are at increased risk for neutropenia compared with both COVID-19-negative patients on clozapine and COVID-19-positive patients not on clozapine. In addition, in the event of inflammation caused by COVID-19, clozapine plasma levels may also rise rapidly. For patients on clozapine who have tested positive for COVID-19 we therefore recommend clozapine dose decreases and more frequent blood counts and clozapine TDM than usually during an infection, even if they have normal body temperature.

Another important symptom in COVID-19-positive patients is respiratory distress. Clearly, high doses of sedatives are unwanted, given their potential respiratory side-effects, such as hypoventilation. Ensuring that the current dose of anxiolytics/hypnotics/sedatives is as low as possible whenever the situation allows and the patient agrees will allow fellow clinicians to better judge respiratory distress and taper such medication when a COVID-19-positive patient is in need.

## Keeping track of possible drug shortages

Fifth, we think that psychiatrists should be mindful that the current global crisis may lead to medication shortages. In recent years, the world has become more reliant on India and China for active pharmaceutical ingredients. Especially now that India has imposed strict social distancing regulations, the supply chain may become compromised.^7,8^ Psychiatrists and clinical pharmacists should follow these developments closely and have a plan in place if shortages occur, for example starting an alternative treatment for a given patient, such as online psychotherapy. Another example from The Netherlands comes from the National Society for Psychiatry, which is in touch with the Ministry of Health and pharmacies on a weekly basis to follow up on possible shortages. During almost weekly seminars and through information on the Society's website, mental health staff can follow such developments closely. We also note that an ethically sound and practical method exists for allocating drugs when shortages arise.^9^

## Conclusions

In sum, we signal several considerations in the current COVID-19 pandemic for physicians prescribing psychotropic compounds. We believe that most of these considerations are generalisable to other countries, as in many countries, including The Netherlands, some mental healthcare services have closed while others have remained open. However, owing to lack of data it is hard to obtain exact figures on access to healthcare facilities across the globe during the current pandemic.

As psychiatrists, general practitioners, nurse practitioners and clinical pharmacists, we should have these considerations in mind when we try to safeguard patient care. We should therefore actively exchange recommendations on safe prescribing of psychotropic medication and alternative strategies (e.g. online psychotherapy) during the current crisis. The Netherlands Psychiatric Association and the UK's Royal College of Psychiatrists recently set up such resources, which are updated frequently with news about the impact of COVID-19 on psychiatry.^10,11^ Given the particularities that apply to each nation, we encourage psychiatrists and clinical psychologists in other countries to do the same. Exchange of clinical experience will also enable clinicians to identify situations where psychological treatments may be preferable. Conceivably, for example, certain cognitive–behavioural interventions may result in more sustained relief of COVID-19-related anxiety compared with psychopharmacological treatment (such as short-term use of benzodiazepines). We also propose that physicians with diverse backgrounds (including GPs, psychiatrists and intensive care doctors) develop prescribing guidelines for important aspects of this COVID-19 outbreak for the field of psychiatry.
